# A Teaching University: A New Hope for London Medicine

**Published:** 1895-02-02

**Authors:** 


### The Hospital

AN INSTITUTIONAL JOURNAL OF

The Medical Sciences and Hospital Administration,

WITH

AN EXTRA NURSING SUPPLEMENT.

Vol. XVII.—No. 436.] [Feb. 2, 1895.

### A Teaching University: A New Hope for London Medicine.

A hope begins to loom large before us that in the near future the medicine of London will establish its right to a great Imperial position. The parochial reactionaries who last year constituted the majority of Convocation of the University of London have reconsidered their decision, and with a public spirit worthy of so great an occasion have resolved that, so far as they are concerned, London shall have a university in fact as well as in name. The specialisation of our functions in journalism compels us to look at the matter almost exclusively from the medical point of view. But no man who has known the experience of breasting the waves of the great open ocean of London life for many years can feel anything but a sentiment of profound satisfaction that London is at last to take her concrete place in the very front rank of those educational pioneers who lead the van of the intellectual progress of the world. London without a university, London practically dumb, has, nevertheless, made herself powerfully felt in every remotest region of our vast dominions. If London, without an intellectual and scientific tongue, has been able to accomplish so much, what may she not hope to effect when she shall have gathered to one great imperial centre of learning and science all the most accomplished voices of our world-wide empire? We cannot resist the temptation, before passing to the consideration of the more limited aspect of the case which medical duty has forced upon us, to express a hope that as London is, and has long been, the greatest mother city of which human history has hitherto had any knowledge, and as she has waited with a patience unshared by any other metropolis for the establishment of an imperial seat of learning in her midst, so she may now, with a greatness worthy of herself, her history, and the unparalleled empire of which she is the centre, establish for herself an imperial university which shall prove a mother to the learning of the world.

Medicine, in the persons of her more philosophic thinkers, already begins to hold up her head, and to scent the fresh breezes of a new day. Too long has the parochial spirit, engendered by an almost exclusively hospital life and experience, ridden, like an old man of the sea, upon the giant back of the philosophic, free breathing, and human medicine which is the creation of the latter half of the nineteenth century. If, as we ardently hope, the phase of mere hospital culture and experience is now nearing its limits, every medical man of academic status and philosophic instincts may begin to breathe more freely.

On such a subject as this, when one essays to descend to details, who will tell us where to begin? In the course of a fight extending over a considerable number of years all the forces massed on the two opposing sides of examination without teaching, and teaching with examination, have used each its utmost skill with the object of winning a victory which should be decisive and permanent. For many years the examinationists pure and simple seemed to be carrying all before them. But, if ever it is proper to say that a thing has been "found out," it is true now to say that examination without teaching has been found out to the very root and core. The teacher, not the examiner, is the one need of the moment. It may be true that when the examining board, which has hitherto gone by the name of the London University, was first established, the stern cracking of the examiner's whip was the thing needed to make lazy students work. But all that we have changed long ago. The lazy student is almost as extinct as the dodo. To-day the average student treads upon the heels of the teacher so eager is he in the pursuit of knowledge, and for the rewards which knowledge brings. Such a student does not need the whip and the spur. It is the rein, the control, and the guidance of larger experience which he requires. These can be furnished by men of University culture alone.

Speaking from a medical standpoint, we cannot but express the conviction that the average medical man of London and the home counties has been hardly treated by the body which has hitherto been called the London University. He has had set before him a very high standard of examination, but in very few instances indeed has there been at his disposal teaching of a commensurate depth and thoroughness. The examination grapes have been good, but they have been placed altogether beyond the reach of any but the very nimblest of medical foxes. That this is not as it should be can be made clear by a single proposition. For an average man an average University degree should be available. For a higher type of intellectual capacity an honours degree should be the object to be aimed at. But the University of London has had nothing but an honours degree to set before London students of all intellectual types and capacities. That surely has been both an injury and an insult to the average medical man. For average men, after all, constitute the bulk of mankind, among doctors as among other classes; and to say that these shall have no rewards at all because they cannot attain to the very greatest is not only unphilosophical but is injurious in the highest degree to the spread of general and scientific culture among the masses of the people.

There is yet another point which is worthy of the most serious consideration on the part of all who take a practical interest in the establishment of a teaching university. London ought to have great positions to offer to great teachers. She should, indeed, have the greatest positions to offer to the greatest teachers. But where are those positions? Do we think of King's College or University? Have any of the London hospitals great positions to offer to great permanent teachers? The University of Edinburgh pays her medical professors on an average five times the amount paid to the average medical teacher at any of the London hospitals. Thus it happens, that in spite of the manifold inducements which London offers to men of teaching capacity to spend their days in her midst more and greater teachers go from metropolitan London to provincial Edinburgh than come from provincial Edinburgh to metropolitan London. In this article we have but faintly outlined the main features of a great subject. Let us hope that every man of culture in London, and, indeed, throughout the British Empire, will make it a matter of personal concern to help forward a scheme for the establishment of a truly metropolitan and Imperial university for the greatest mother city in the world.

